# Performance and Long Term Stability of Mesoporous Silica Membranes for Desalination

**DOI:** 10.3390/membranes3030136

**Published:** 2013-07-12

**Authors:** Muthia Elma, Christelle Yacou, João C. Diniz da Costa, David K. Wang

**Affiliations:** FIMLab—Films and Inorganic Membrane Laboratory, School of Chemical Engineering, The University of Queensland, Brisbane, Queensland 4072, Australia; E-Mails: m.elma@uq.edu.au (M.E.); c.yacou@uq.edu.au (C.Y.); j.dacosta@uq.edu.au (J.C.D.C.)

**Keywords:** mesoporous, silica, membrane, sol-gel, desalination, stability

## Abstract

This work shows the preparation of silica membranes by a two-step sol-gel method using tetraethyl orthosilicate in ethanolic solution by employing nitric acid and ammonia as co-catalysts. The sols prepared in pH 6 resulted in the lowest concentration of silanol (Si–OH) species to improve hydrostability and the optimized conditions for film coating. The membrane was tested to desalinate 0.3–15 wt % synthetic sodium chloride (NaCl) solutions at a feed temperature of 22 °C followed by long term membrane performance of up to 250 h in 3.5 wt % NaCl solution. Results show that the water flux (and salt rejection) decrease with increasing salt concentration delivering an average value of 9.5 kg m^–^^2^ h^–1^ (99.6%) and 1.55 kg m^–^^2^ h^–1^ (89.2%) from the 0.3 and 15 wt % saline feed solutions, respectively. Furthermore, the permeate salt concentration was measured to be less than 600 ppm for testing conditions up to 5 wt % saline feed solutions, achieving below the recommended standard for potable water. Long term stability shows that the membrane performance in water flux was stable for up to 150 h, and slightly reduced from thereon, possibly due to the blockage of large hydrated ions in the micropore constrictions of the silica matrix. However, the integrity of the silica matrix was not affected by the long term testing as excellent salt rejection of >99% was maintained for over 250 h.

## 1. Introduction

Water scarcity is a serious global issue that is exacerbated by increasing global population, rising seawater level and disappearing freshwater aquifers and reservoirs. Literally, around 97% of the world’s water exceeds the potable water quality which cannot be consumed or even be used for agricultural purposes [1,2]. Hence, sourcing potable water from sea water, a process called desalination, is a logical way ahead to address the water requirements of the current and future needs of our society. Desalination of salty waters (brackish, seawater and brine water) can be carried out by membrane separation processes such as reverse osmosis (RO), membrane distillation (MD) or pervaporation (PV). In particular, RO using polymeric membranes are now the most widely applied membrane separation technology for desalination around the world. For instance, large desalination plants have been built in Spain, Australia, Saudi Arabia and United Arab of Emirates [3]. Although RO is the most energy efficient process for water desalination to date, it relies on a pressure-driven solution-diffusion process of separating fresh water via a dense membrane which often suffers from polymer swelling, biofouling, scaling and poor thermal and chemical resistance [4,5].

Inorganic membranes have recently attracted the attention of the research community and industry owing to robustness, long life-span and better resistance to extrinsic environmental and industrial factors. Currently, there are two types of widely researched inorganic silica membranes for desalination, namely zeolites [6,7,8] and amorphous silica membranes [9,10,11]. Zeolite membranes have proven to be effective but their long term stability and scale up still present an on-going issue [8]. Silica membranes have excellent molecular sieving properties and a simpler fabrication process via sol-gel processing, though they need functionalisation to overcome hydro-instability. The structures of silica membranes have pore sizes in the range of 3–5 Å on the order of the kinetic diameter of the water molecule (d_k_ = 2.6 Å), thus ideal to hinder the passage of hydrated salt ions (e.g., Na^+^: d_k_ = 7.2 Å and Cl^–^: d_k_ = 6.6 Å). Therefore, silica membranes separate water from salts by a molecular sieving mechanism [12]. Overall, the interest in amorphous silica based membranes derived from sol-gel is gaining research momentum particularly the effort is emphasized on improving their membrane performance and hydrothermal stability [10].

Owing to the affinity of these membranes for water adsorption which leads to structural degradation and a loss of selectivity, several research strategies have been employed to overcome hydro-instability. These include carbonized templating [9,13], metal doping [11,14,15,16,17,18] and hybrid organosilica [19,20,21] methods. These strategies concertedly attempt to modify the surface properties of silica in order to minimize the interaction of water molecules with the surface silanol groups (Si–OH). However, the water fluxes reported by these works are generally too low and research in this area is still at its infancy. In order to address these problems, it becomes important to reduce the level of silanol concentrations in the silica matrix to produce an improvement in the structural stability and water flux. In addition, these silica base membranes have been prepared by acid catalyzed sol-gel method with low pH of generally below 2.5. At these conditions, amorphous silica structures with molecular sieving domains are formed, though the sol-gel synthesis favours the production of silanol species.

In this work we modified the sol-gel process to reduce the amount of silanol groups. This was achieved by a two-step sol synthesis, where the first step followed a traditional acid catalysis and the second step included pH modification using ammonia base catalysis to favour the condensation of silanols to siloxane bridges (Si–O–Si). The latter is known to oppose hydro-instability in silica membranes [16,22,23]. To demonstrate this concept, silica films were coated on alumina substrates and membranes were tested. The morphology and long term operation and stability are reported along with the xerogels characterization by FTIR and N_2_ sorption.

## 2. Experimental Section

### 2.1. Materials and Membrane Synthesis

Silica sols were synthesized by a two-step sol-gel process employing acid and base as catalysts. Tetraethyl orthosilicate (TEOS, 99.0%, (GC) Sigma-Aldrich) was added drop-wise into ethanol (EtOH, 99%) and stirred for 5 min in ice bath condition at 0 °C to avoid partial hydrolysis followed by the addition of diluted nitric acid (0.0008 M HNO_3_, Merck). The sol mixture was refluxed for 1 h at 50 °C under vigorous stirring to achieve a complete hydrolysis of the alkoxy groups. Ammonia solution (NH_3_, 25%, Merck) diluted in ethanol was added drop wise into sol mixture to commence the condensation reaction and reflux was continued for another 2 h to obtain the resultant sol which was then dried in a temperature controlled oven at 60 °C for 24 h to obtain the dried gel. The final molar ratios of the TEOS:EtOH:HNO_3_:H_2_O:NH_3_ sol were calculated to be 1:38:0.0007:5:*x*, where *x* was varied from 0.003, 0.008, 0.02 and 0.08 to prepare the final sol pH of 6 to 9 ± 0.1. The dried gel was grounded into powder and calcined in a temperature controlled furnace in air at 600 °C for 4 h with 1 °C min^–1^ ramping and cooling rates.

Thin membrane films were coated on macroporous alumina substrates (α-Al_2_O_3_ tubular support (Φ_pore size_ ≈ 100 nm), Ceramic Oxide Fabricates, Australia) using the resultant sol prepared at pH 6 via a dip-coating process with a dwell time of 2 min and a dipping and withdrawal rate of 10 and 5 cm min^–1^, respectively. After the deposition of each layer, the membrane layer were dried briefly in an oven and then calcined in a furnace according to the same treatment described above for the xerogels. This cycle of dip-coating, drying and calcination was repeated four times for a total of four membrane layers.

### 2.2. Materials and Membrane Characterisation

Nitrogen physisorption analysis at 77 K and 1 bar were conducted using Micromeritic TriStar 3020 instrument. Sample was degassed under vacuum for >6 h at 200 °C. The specific surface area was determined from Brunauer, Emmett and Teller (BET) method and total pore volume was taken from the last point of the isotherm. Dubinin-Astakhov and Barrett-Joyner-Halenda methods were taken to determine the average pore sizes of microporous and mesoporous materials, respectively. The analysis of pore size distribution was obtained using the density functional theory (DFT) method for pore sizes between 1 to 50 nm for micropore and mesopore range [24,25,26,27]. Fourier Transform Infra-Red (FTIR) spectra data were collected at a resolution of 4 cm^–1^ in the range of 4000–600 cm^–1^ for a total of 30 scans using Shimadzu IR affinity-1 with a Pike MIRacle ATR attachment. Peak deconvolution of the absorption bands over the region 1300–600 cm^–1^ was performed with FityK software using Gaussian lineshapes with the least square fit routine [28] and peak areas were measured for the normalized spectra using a local baseline. The membrane morphology and thickness were characterized by field emission scanning electron microscopy (FESEM JOEL 7001).

### 2.3. Membrane Desalination and Long Term Stability

**Figure 1:**
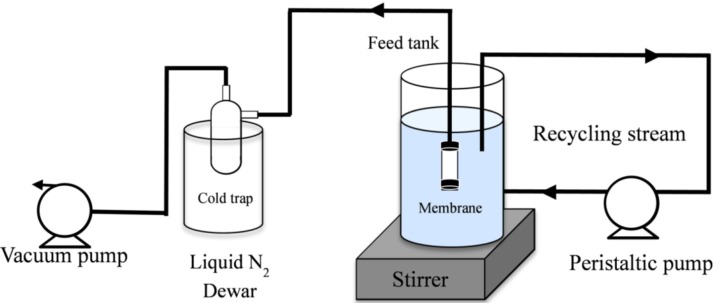
Desalinating rig using a customised pervaporation set up.

The membrane was assembled into a classical pervaporation set-up for desalination experiments as shown in Figure 1. Briefly, the membrane tube was blocked at the bottom side (dead end mode) and immersed in the tank containing a saline feed solution which was open to atmospheric condition. The temperature (22 ± 1 °C) of the feed solution was maintained at room temperature and monitored by a thermometer. The feed tank was then connected to a peristaltic pump where the retentate stream was constantly recycled and stirred to prevent concentration polarization on the feed side of the membrane. The membrane was connected to a vacuum line and the permeate stream was collected in a cold trap which was immersed in a liquid nitrogen dewar.

To prepare the saline feed solution, the powder of sodium chloride (NaCl, Sigma Aldrich) were dissolved in deionised water to prepare the salt solution concentrations ranging from 0.3 to 15 wt %. The water flux, F (kg m^2^ h^−1^), was determined based on the Equation F = *m*/(*A*·Δ*t*), where *m* is the mass of permeate (kg) retained in the cold trap, *A* is the surface-active area (m^2^) and Δ*t* is the time measurement (h). The salt rejection, R (%), was calculated as R = (*C_f_* − *C_p_*)/*C_f_* × 100%, where *C_f_* and *C_p_* are the feed and permeate concentrations of salt (wt %). Conductivities of the retentate and permeate solutions were determined by using a conductivity meter (labCHEM CP). Lastly, long term membrane performance for hydrostability was conducted in a 3.5 wt % feed NaCl solution at 22 °C.

### 2.4. Error Analysis

The data represents the mean of six permeate sample solutions from two identical membranes with two standard deviations from the mean representing a 95% confidence interval.

## 3. Results and Discussion

### 3.1. Xerogel Characterisation

**Figure 2:**
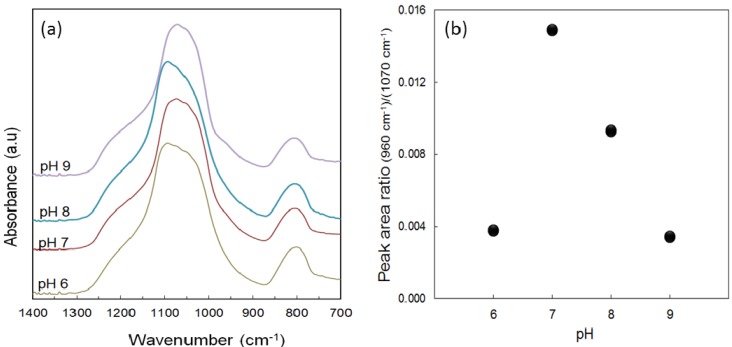
(**a**) FTIR spectra of the calcined silica xerogels and (**b**) the peak area ratio of the silanol peak at 960 cm^−1^ against the siloxane peak at 1070 cm^−1^ for the spectra as a function of the sol pH.

The FTIR spectra of the calcined xerogel samples in Figure 2a show very similar vibrational bands in the region of 1400–700 cm^−1^ for all samples independently of their pH. First of all, the intense peak near 1070 cm^−1^ along with bands of lesser intensity at 1160, 1030 and 800 cm^−1^ are all assigned to various stretching and bending vibrations of the siloxane (Si–O–Si) groups [29]. The other peak of interest appearing at the peak at 960 cm^−1^ is attributed to the vibrational stretching of the silanol (Si–OH) groups. A scan of the spectral profiles also suggests that the chemical constituents are quite similar in all the samples. The change of the vibrational bands relating to the silanol and siloxane concentration was quantitatively assessed by a deconvolution of the bands at 960 and 1070 cm^−1^, respectively. The peak area ratio analysis regarding the silanol *versus* the siloxane groups is presented in Figure 2b. The results show that this ratio increases as pH is increased but then decreases again at pH 8 down to a minimum observed at pH 9. This behaviour could be explained on the basis of the pH-dependency of the hydrolysis, condensation and polymerization reactions for TEOS system as has been reported extensively by Brinker *et al*. [30,31,32]. These results clearly indicate that the lowest silanol concentrations, or likewise the highest siloxane bridge concentrations, were achieved with calcined xerogels prepared with pH 6 or 9.

In a two-step, acid-base, sol-gel process, the first step was carried out under an acidic condition (pH ~ 4) under reflux. Acid catalyzed hydrolysis with heating promoted a high production of silanol species from the silane precursor. When pH of the sol is adjusted by the addition of the ammonia hydroxide in the second step, the sol pH increases rapidly to greater than 4, which is much higher than the isoelectric point boundary (pH 1–3) of the silica species [30]. At this instance, the silanol species are expected to be all deprotonated participating in the polycondensation reaction generating a large concentration of highly condensed species (e.g., siloxane bridges) as shown by the lower peak ratio in the case of pH 6 (Figure 2b). At near neutral pH of 7, there appears to be another boundary because of the solubility and dissolution of the formed silica are maximized as silica is much more soluble in alkaline solutions than in acidic ones [30,31]. In this sample, hydrolysis occurs preferentially on the monomers and the weakly branched oligomers that subsequently condense preferentially with the silica clusters leading to a higher degree of uncondensed silanol species [30], and this is indicated by the highest peak area ratio for this sample. The mechanism of structural formation in this case is strongly governed by a monomer-cluster growth that favours a more compact structure. Above the pH 7, polymerization typically occurs via Ostwald ripening. Particles grow even more rapidly under the refluxing condition and the weakly cross-linked polymers formed in the first hydrolysis step tend to be dissolved and then re-precipitate on the more highly condensed sites [31,32], as this was observed by a fast onset of gelation in the pH 9 sample. Hence, the observed silanol/siloxane ratio decreases from pH 7 thereafter.

**Figure 3:**
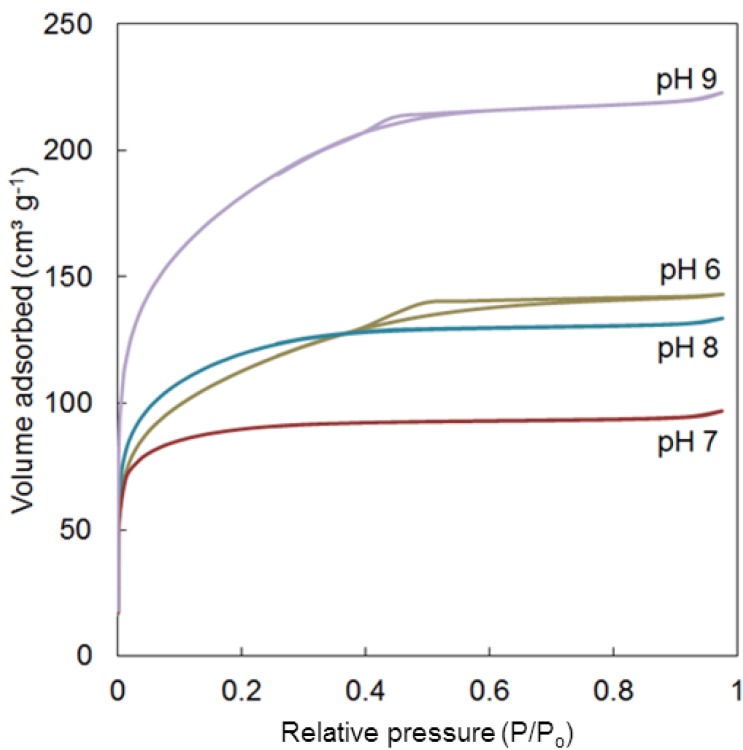
Plot of N_2_ sorption isotherms for the calcined xerogels.

The surface and pore properties of the bulk silica xerogels were analyzed by N_2_ sorption technique which provides important qualitative information regarding the microstructure of the resulting molecular sieving membranes. In general, pore sizes with diameters greater than 50 nm are classified as macroporous; between 2 and 50 nm are mesoporous and smaller than 2 nm are microporous. The N_2_ sorption isotherms for the xerogel samples are shown in Figure 3 and BET surface areas, total pore volumes and average pore diameters are listed in Table 1.

**Table 1:** Surface properties of the bulk silica xerogels.

Sol pH	BET surface area (m^2^ g^−1^)	Total pore volume (cm^3^ g^−1^)	Average pore diameter (nm)
6	402	0.221	2.70
7	415	0.150	1.76
8	425	0.207	1.93
9	649	0.345	2.60

The isotherm profiles of the xerogels prepared at different sol pHs show a very different behaviour even though various proportions of microporosity/mesoporosity are present in all the samples. Xerogels prepared in pH 6 and 9 show a tendency to form micro and mesoporous materials as the adsorption saturation was achieved above 0.65 P/P_0_ with the capillary condensation leading to hysteresis near 0.5 P/P_0_. Their respective average pore diameters are measured around 2.6–2.7 nm. These results corroborated with a higher amount of siloxane bridges shown in Figure 2b. On the other hand, samples prepared at pH 7 and 8 produced a Type I isotherms with no hysteresis, characteristics of a typical microporous material. Their BET surface areas (~420 m^2^ g^−^^1^) and total pore volumes (~0.18 cm^3^ g^−^^1^) are quite comparable with pore sizes of around 1.8 nm. Hence, microporosity correlates well with a high concentration of silanol groups, and in line with previous reports on silica membranes [11,18].

### 3.2. Membrane Morphology

**Figure 4:**
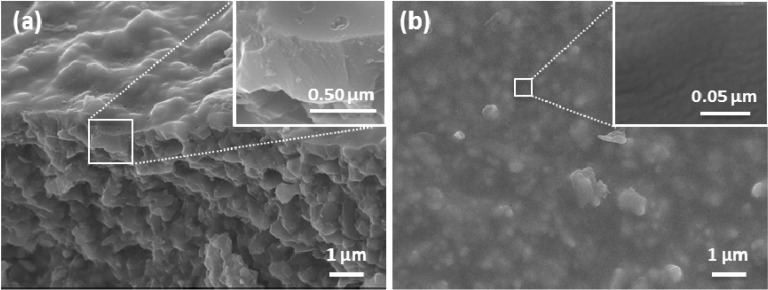
SEM images of the as-synthesized pH 6 sol-gel derived membrane in (**a**) cross-section and (**b**) top surface with the inset images showing the close up of the respective morphology.

Figure 4a,b display representative scanning electron microscope images of a synthesized silica membrane and the underlying alumina substrate. The cross section of the membrane shows that there is a lack of clear distinction between the top layer and the macroporous substrate layer. Without the intermediate layer, the top layer sol is expected to infiltrate deep into the macropores of the substrate where the intermingling between the two layers is unavoidable. The thickness of the silica layer is estimated to be ~470 nm as shown by the inset of Figure 4a. In fact, as evidenced by Figure 4b, the surface morphology of the membrane clearly shows that the roughness of the macroporous alumina substrate is translated onto the silica layer which basically followed the morphology of the substrate whereby the grain size of the α-Al_2_O_3_ particles is estimated to be approximately 0.5 µm. Despite this, the surface morphology of the silica layer in the inset of Figure 4b appears to be homogeneous without any major cracks or defects that would otherwise contribute to the poor membrane performance.

Conventionally silica films are coated on γ-Al_2_O_3_ interlayers (small pore sizes of 3–5 nm) instead of substrates with α-Al_2_O_3_ (larger pore sizes of 100–300 nm) [33,34,35]. This is the general approach for coating acid catalysed silica films [11,36,37], particularly to avoid cracking or defects in the top silica films. By changing the silica sol-gel synthesis, this work demonstrates for the first time that defect free silica films can be directly coated onto a macroporous substrate without any intermediate layer. This coating strategy is attractive as it reduces the number of layers—at least two interlayers are no longer required—and consequently reducing preparation time and production costs.

### 3.3. Membrane Performance and Long Term Stability

**Figure 5:**
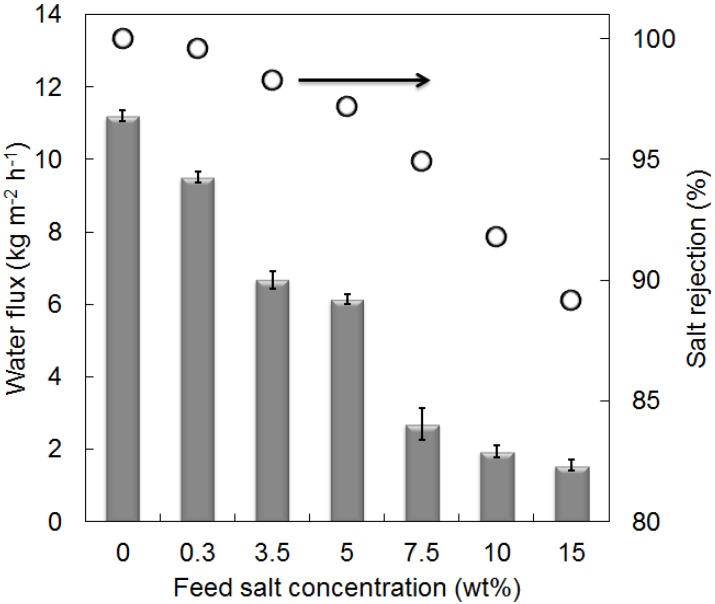
Membrane desalination performance in pervaporation mode as a function of the feed salt concentrations at 22 °C. The water fluxes are shown in the vertical bars (left axis) and the salt rejection values are shown in the unfilled circles (right axis).

From these preliminary results and analyses, it was shown that the sample prepared by the pH 6 and 9 sol produced a relatively lower concentration of the surface hydrophilic silanol species. The pH 9 sols precipitated very quickly, followed by gelation. Hence, pH 9 sols were unsuitable for membrane preparation. Therefore, pH 6 was chosen to directly dip-coat the macroporous α-Al_2_O_3_ substrate without further priming with intermediate layers. Membrane performance in terms of water fluxes and salt rejections was evaluated in various salt feed concentrations (NaCl 0.3–15 wt %) at 22 °C as shown in Figure 5. The membranes were initially tested in pure water followed by increasing salt concentrations in order to simulate brackish (0.3 wt %), sea (3.5 wt %) to brine (>5 wt %) waters. In Figure 5, the membranes delivered considerable high water fluxes of 9.5 kg m^−2^ h^−1^ and excellent salt rejections in excess of 99% for brackish NaCl 0.3 wt % concentration. The membrane performance decreased as a function of the salt concentration reaching a minimum of 2.8 kg m^−2^ h^−1^ (95% salt rejection) and 1.6 kg m^−2^ h^−1^ (89%) for the brine feed waters 7.5 and 15 wt %, respectively.

The reduction of water fluxes with increasing the feed salt water concentration is mainly associated with the polarisation effect. As water permeates through the membrane, its concentration on the membrane surface reduces, consequently the concentration of salt increases on the membrane surface. This salt polarisation effect is controlled by chemical equilibrium, as an increase in salt at the membrane surface will result in water diffusing from the bulk feed solution to the membrane surface. Hence, the salt polarisation effect tends to limit the driving force for water permeation. A secondary effect is the blocking of pore size constrictions by large hydrated salt ions [11]. This is important as the xerogel samples in Figure 3 resulted in micro and mesoporous materials. Hence, percolative pathways in the amorphous matrix may be blocked by the larger hydrated salt ions.

In addition, the reduction of salt rejection as the salt feed concentration is closely associated with both salt polarisation effect and the structure of the pH 6 silica membrane. As the feed salt concentration increases at the membrane surface, so does the polarisation effect. As a result, the driving force for salt diffusion increases across the membrane. The pH 6 silica membrane also has mesopores as per Figure 3. If mesopores are linked together in the amorphous silica matrix, then there is a preferential percolative pathway favouring hydrated salt diffusion. Figure 5 shows that this is the case as salt was measured in the permeate stream, though the salt rejections were high. Hence, these results suggest that the concentration of mesoporous percolative pathways through the amorphous silica film is small.

The membranes prepared in this work performed favourably as compared to previous inorganic membranes reported in open literature as listed Table 2. For similar testing conditions (NaCl 3.5 wt %; 22 °C), it is observed the pH 6 silica membrane (6.8 kg m^−2^ h^−1^; >98% salt rejection) delivered a water flux 20 fold higher than cobalt oxide silica membrane (0.35 kg m^−2^ h^−1^; >99.5%) [11], and at least 2 fold higher than microporous carbonized templated silica membranes (1.9 kg m^−2^ h^−1^; >98%) [9] and mesoporous hybrid organosilica membranes (3 kg m^−2^ h^−1^; >99.5%) [19,21], whilst salt rejections were high and comparable. In this body of work [9,11,19,21], the membranes were prepared via an acid-catalysed sol-gel process with relatively low pH, as a result of which forms microporous silica thin films. Therefore, these membranes naturally produced lower water fluxes due to a higher concentration of microporous percolative pathways.

Remarkably, the ability of this membrane to process brine conditions of up to 15 wt % feed is very attractive with a much higher water flux than that is reported for metal oxide silica membrane [11], albeit with a lower rejection rate which can be overcome by a second pass filtration and/or further membrane development. One possible explanation for the higher fluxes produced by the membranes in this work could be that they were prepared without any intermediate layer which can also contribute to the water transport resistance, unlike the other reported membranes with several intermediate layers. Furthermore, when compared to zeolite-based membranes, the membrane performance of MFI-ZSM-5 [8] produced similar high water fluxes at low salt concentrations but trended oppositely as salt concentration increases. Despite that the MFI-ZSM-5 membrane produced much higher fluxes at 15 wt % feed, though the salt rejection was severely compromised due to poor membrane stability and dissolution of the charged double layer in the zeolitic pores. The major advantages of pH 6 silica membrane are better desalination performance at mild testing conditions.

**Figure 6:**
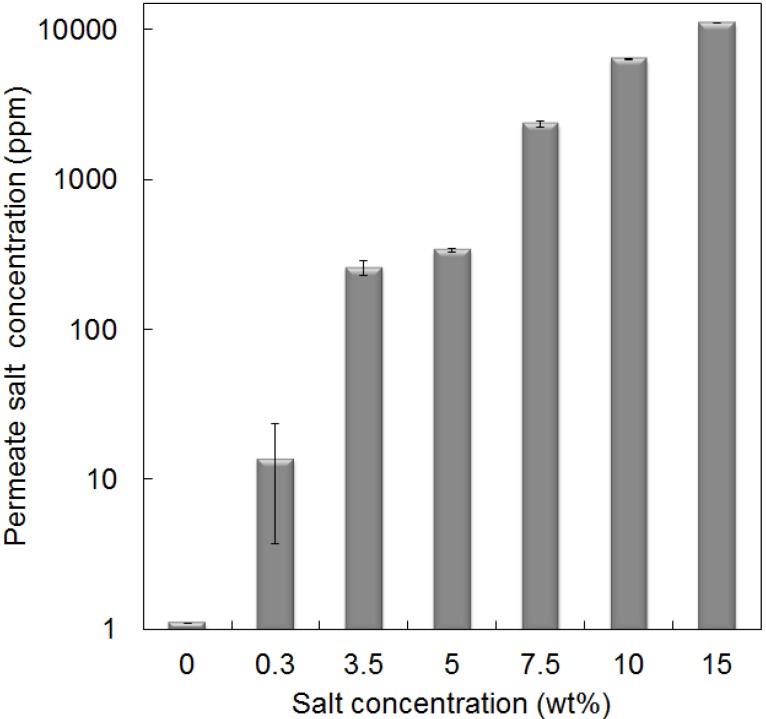
Permeate salt concentration as a function of the feed salt concentration at 22 °C.

The purity of the permeate water was assessed by evaluating the salt concentration (parts per million) calculated from a standard curve based on the conductivity measurements. Figure 6 demonstrates that the permeate salt concentration was calculated to be 220 and 340 ppm for 3.5 and 5 wt % feed solutions, respectively. These are well below the recommended concentration of 600 ppm (total dissolved salt) for potable water according to the World Health Organization [1]. These results clearly show that the silica membranes prepared in this work can be used to treat saline waters across a broad range of salt concentrations at the room temperature conditions with excellent performance.

**Table 2:** Performance comparison of silica based membranes for desalination.

Membrane type		Feed Temp. (°C)	Feed conc. range (wt %)	Water flux (kg m^–2^ h^–1^)	Rejection (%)	Reference
**Pure silica**	TEOS pH 6	22 ^b^	0.3 3.5–15	9.5 6.8–1.6	99.6 98–89	This work
**Carbonized template**	Ionic C6	20 ^a^	0.3–3.5 *	2.1–1.9	99.9–98	[9]
	Ionic C16	20 ^b^	0.3–3.5	3.0–2.0	91–97	[13]
	20 wt % PEG-PPG	20 ^b^	0.3–3.5	6.3–4.9	87–97	[38]
**Metal oxide**	CoOxSi	20	0.3–15	0.4–0.3	99.7–99.9	[11]
**Hybrid**	BTESE	30	0.2	3.0	99	[21]
	MTES	20 ^a^	0.3–3.5 *	4.7–2.5	93.7–83	[9]
**Zeolite**	MFI-S-1	25	0.5	0.12	75	[39]
	MFI-ZSM-5	25	0.5	1.1	93	[40]
	MFI-ZSM-5	22	3.8 *	0.70	99	[41]
	MFI-ZSM-5	20	0.3–15	4.5–9.8	99.5–74	[8]

^a^ Feed pressurizing up to 7 bar and permeate vacuum pumping; ^b^ Pressure difference (ΔP) of <1 bar across the membrane; * Sea water.

**Figure 7:**
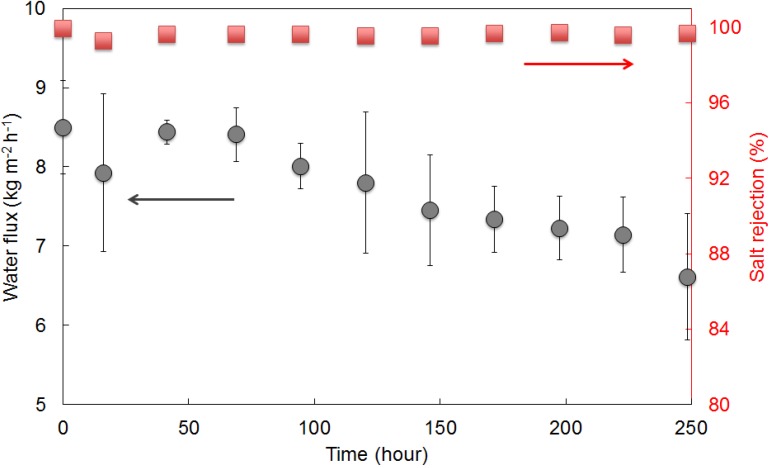
Water fluxes and salt rejection of the membrane as a function of exposure time over 250 h in 3.5 wt % feed saline solution at 22 °C.

One major concern of the membrane performance is their long term stability. Long term membrane operation and stability was conducted using NaCl 3.5 wt % condition at 22 °C after the membrane desalination tests. As can be seen from Figure 5 that both flux and rejection have dramatically declined after the membranes were tested in 15 wt % feed solution (1.6 kg m^−2^ h^−1^, 89%). As a result, the membranes were washed with a copious amount of water to ensure that any salt residues and blockages were removed. Figure 7 shows the membrane water flux and salt rejection over the 250 h of test period. Preliminary stability results demonstrate that the membranes were reasonably stable for the first 150 h with water fluxes of approximately 8.5 kg m^−2^ h^−1^ after which a gradual decay in the fluxes had occurred reaching 6.7 kg m^−2^ h^−1^ for the 250 h test. The reduction in water fluxes could be attributed to the blocking of hydrated salt ions in microporous constrictions as discussed above.

Interestingly, the flux at initial times (Figure 7) was measured slightly higher than the flux recorded previously as shown in Figure 5 after the washing procedure. The results of the long term stability suggest that the membrane matrices may be undergoing textural structural changes and/or a build-up of salt residues causing pore blockages. Nevertheless, this long term testing successfully demonstrated that the membranes are still capable of separating saline water at this treatment condition. Of particular attention, the integrity of the pH 6 silica film was not affected, as salt rejections remained greater than 99% at all times.

**Figure 8:**
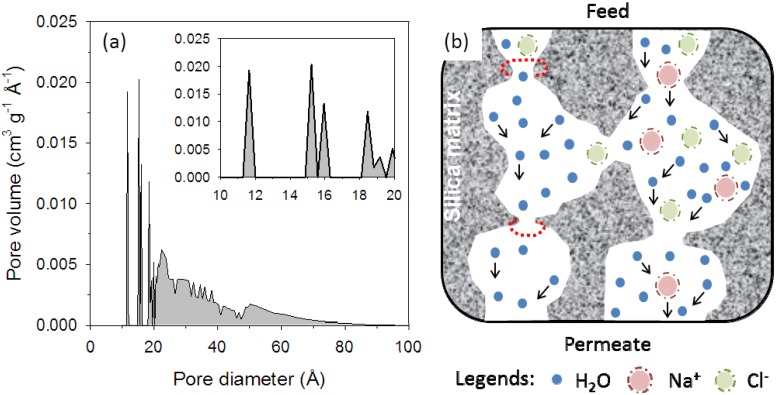
(**a**) Pore size distribution the xerogel sample using the density function theory method with inset displaying between 10 to 20 Å and (**b**)Schematic illustrating percolative porous pathway and diffusions of water and hydrated salt ions through the pores of the silica matrix with the red halos representing the location of the microporous constrictions.

In this work, the membrane was clearly able to provide a selective barrier between the water molecule and the hydrated salt ions. However, as shown by the N_2_ isotherm (Figure 3), this membrane material pH 6 silica has a combination of micro and mesoporous texture with an average pore size measured at 27 Å, which is obviously much larger than the kinetic diameter of the permeating salt ions in this study. To further understand these results, Figure 8a displays the pores size distribution (PSD) of this membrane material (calcined bulk xerogel) based on the N_2_ isotherms discussed above. The PSD consisted of both microporous (<2 nm) and mesoporous pores (2–10 nm). It is important to bear in mind that the properties of deposited thin films may be quite different from bulk xerogels due to non-equivalent gelation and drying conditions [42]. Even though the properties of the xerogels and the thin-layer films are non-equivalent, the membrane materials in the form of xerogels are often used as a qualitative comparison of pore size distribution and pore evolution [17,18,25,43,44] due to the ease of sample synthesis and characterization.

To further assess the performance of the membranes, Figure 8b shows a schematic of the pH 6 silica films in this work. Essentially silica films have smaller pore sizes than the bulk xerogel samples, which is evidenced by the higher salt rejection (Figure 5, Figure 7). If the silica pores were all mesoporous, then it is very likely that the membranes would not be able to reject the salt ions. It is known that amorphous silica possess a trimodal PSD consisting of small 3 Å and large 8 and 12 Å pore sizes as determined by positron annihilation spectroscopy [45]. Therefore, the membrane results in this work suggest that the salt rejection into the amorphous pH 6 silica membranes was controlled by a percolative pathway containing constrictions below the size of hydrated salt ions (Na^+^: d_k_ = 7.2 Å and Cl^−^: d_k_ = 6.6 Å) [12,46] and above water (d_k_ = 2.6 Å). However, silica films may also contain percolative pathways composed of mesoporous regions linked to microporous constrictions or to another mesoporous region. In view of the small amount of salt permeated through the membrane, it is evident that a small number of mesoporous percolative pathways (>7.2 Å) were present in the pH 6 silica film.

## 4. Conclusions

Mesoporous silica membranes have been successfully fabricated by dip-coating deposition of silica sol prepared from a two-step sol-gel process. pH 6 condition was chosen, as synthesized sample produced the lowest silanol concentration, a requirement to obtain hydro-stable silica membranes. Hence, pH 6 silica membranes delivered high water fluxes and salt rejection at 9.5 kg m^–2^ h^–1^(salt rejection 99.6%) and 6.8 kg m^–2^ h^–1^ (98.2%) for the 0.3 and 3.5 wt % saline solutions, respectively. Furthermore, the membranes can effectively produce potable water in these conditions achieving permeate salt concentration of less than 600 ppm. In addition, the tested membrane was reasonably stable for the first 150 h after which a gradual decay in the fluxes was observed resulting in approximately 6 kg m^–^^2^ h^–^^1^ at the 250 h, albeit with salt rejections, were calculated to be greater than 99% at all times. The flux reduction was attributed to the blocking hydrated salt ions in the constrictions of the micropores in the amorphous silica film. Overall, the membrane performance exceeded the expectations for silica-based membranes reported in the literature thus far.
